# First-principles investigation of the mechanically and thermodynamically stable K_2_TlXCl_6_ (X = Sb or Sc) compounds for energy harvesting and photocatalytic applications

**DOI:** 10.1039/d6ra03926j

**Published:** 2026-07-08

**Authors:** Mubarra Javed, Muhammad Yaseen, Nada Alfryyan, Sidra Sarfraz, Muhammad Adnan, Saima Noreen, Muhammad Zahid, Imed Boukhris

**Affiliations:** a Department of Physics, University of Agriculture Faisalabad Faisalabad 38040 Pakistan myaseen_taha@yahoo.com; b Department of Physics, College of Sciences, Princess Nourah bint Abdulrahman University P. O. Box 84428 Riyadh 11671 Saudi Arabia; c Department of Chemistry, University of Agriculture Faisalabad Faisalabad 38040 Pakistan; d Central Labs, King Khalid University AlQura'a, Abha, P.O. Box 960 Saudi Arabia; e Department of Physics, College of Science, King Khalid University Abha P.O. Box 960 Saudi Arabia

## Abstract

Herein, the physical characteristics of K_2_TlSbCl_6_ and K_2_TlScCl_6_ are explored using the modified Becke–Johnson potential. The structural, optical, and thermoelectric characteristics are substantially impacted by the B′ site ordering. Both compounds are mechanically, structurally and thermodynamically firm, as confirmed by Born's criteria, Goldschmidt's tolerance factor and formation enthalpy, respectively. Electronic structure properties indicate the semiconducting behavior of K_2_TlSbCl_6_/K_2_TlScCl_6_ having energy gaps values of 2.26/3.83 eV, respectively. Mechanical analysis indicates their ductile behavior, with a positive Cauchy pressure, Poisson/Pugh ratio higher than 0.26/1.75, correspondingly. Optical studies reveal high absorption coefficients (*α*(*ω*)) of ≈6.59 × 10^5^ cm^−1^ for K_2_TlSbCl_6_ and 7.13 × 10^5^ cm^−1^ for K_2_TlScCl_6_. The compounds also exhibit low reflectivity and transparency from the visible to ultraviolet regions with static refractive indices n(0) > 1.8. TE analysis further reveals a figure of merit (*ZT*) higher than 0.7 at 300 K. Moreover, band-edge alignment indicates that K_2_TlScCl_6_ is suitable for hydrogen generation. Overall, the calculated TE and optoelectronic features suggest that K_2_TlSbCl_6_ and K_2_TlScCl_6_ are useful for power generation applications.

## Abstract 2

Electronic structure calculations reveal the semiconducting behavior of K_2_TlSbCl_6_/K_2_TlScCl_6_ with band gap values of 2.26/3.83 eV, respectively.

## Graphical abstract

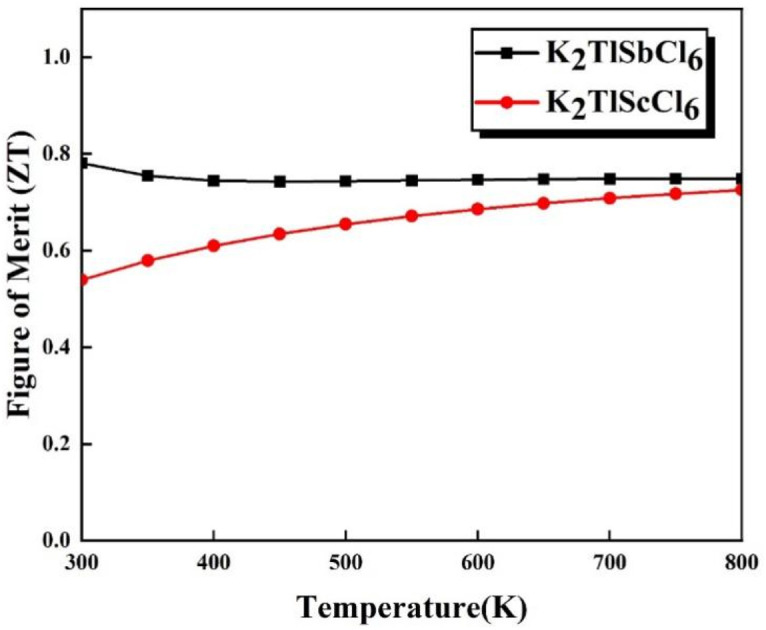

## 1. Introduction

The rising demand for electricity worldwide, primarily met by fossil fuels, has heightened the concerns about environmental degradation and the need for clean alternatives. However, fossil fuel reserves are finite, and their ongoing consumption makes a substantial contribution to global warming and ecological degradation.^1–3^ Among renewable energy technologies, solar cells and TE generators have attracted considerable interest as effective and sustainable solutions to meet the increasing energy demand.^4–6^ In this context, perovskite materials exhibit exceptional physical properties and structural flexibility; a wide variety of elements from across the periodic table is capable of forming stable perovskite phases. Also, these materials possess properties like ferromagnetism, superconductivity, and ionic conductivity, which play significant roles in a wide range of advanced technologies.^7–9^ Moreover, perovskite (ABX_3_) materials exhibit a broad range of interesting physical properties due to their structural flexibility. In the formula ABX_3_, ‘A’ can be +1 organic or inorganic cations, ‘B’ can be any suitable small high valence metal cation, and ‘X’ is usually a oxide, halide, or any other chalcogenide anion.^10,11^ Furthermore, because of their tunable *E*_g_, efficient charge extraction, and great optical absorption, these materials have received a lot of attention for photovoltaic and optoelectronic applications.^12–14^ Despite their tremendous performance, conventional Pb-based halide perovskites (HPs) cause major environmental problems because lead is poisonous. This has prompted extensive research into Pb-free alternatives, specifically halide double perovskite (HDP) materials having A_2_B′B″X_6_ formula. These materials retain desirable optoelectronic properties while eliminating the toxic components. HDPs have shown potential for applications in various technologies, such as solar absorbers, UV detectors, scintillators, and X-ray detectors, due to their better optical, structural, thermal, and electronic characteristics. Importantly, the selection of the B′ and B″ sites components is important in determining whether the material exhibits a direct/indirect *E*_g_.^15,16^ While the A-site cations and X-site halides exert minor influence on the *E*_g_ nature, the B-site configuration, particularly involving B^3+^ cations, is pivotal to achieve a direct and non-toxic semiconductor.^17–20^ Inspired by these fascinating attributes, first-principles computational methods are widely employed to reveal the geometrical, optoelectronic, and TE features of various HDPs, providing valuable guidance for designing reliable and efficient Pb-free HDPs.^21–24^

Several stable and non-toxic lead-free HDPs have been identified for applications in green energy technologies, including solar absorbers, LEDs, and other photovoltaic applications.^25,26^ For instance, the non-magnetic nature of Rb_2_NaCoF_6_ makes it suitable for thin-films and high-performance ultraviolet (UV) optoelectronic devices. Additionally, Rb_2_TlInX_6_ (X = halogen) HDPs have been identified as potential contenders for future photo-electronic systems due to their suitable structural and electronic characteristics.^26^ HDPs such as K_2_TlSbY_6_ (Y = I, Cl, and Br) exhibit a direct *E*_g_ of 0.89, 1.35, and 1.05 eV, respectively, which reveal their optimal range for photovoltaic applications.^27^ Similarly, A_2_TlSbX_6_ (A = alkali metal) HDPs were theoretically predicted to possess a direct *E*_g_ within 1.82–2.76 eV, reflecting their potential use in photovoltaics.^28–30^ These works established the structural firmness, direct *E*_g_ nature, and promising TE operation of the Tl–Sb-based halide double perovskites and positioned them as potential contenders for solar systems and TE conversion systems. However, compositional variations involving trivalent cations beyond Sb^3+^, such as Sc^3+^, remain unexplored, particularly with regard to their influence on band structure engineering, lattice dynamics, and charge transport. In this context, the present work provides new insights by systematically comparing K_2_TlSbCl_6_ and K_2_TlScCl_6_ to understand how substituting Sb with Sc, which has a lower ionic radius and different electronegativity, modifies the *E*_g_, effective masses, and optical absorption. Furthermore, their geometry and optoelectronic and TE characteristics are calculated for their prospective application in multifunctional energy devices.

## 2. Computational work

DFT calculations were executed to determine the physical features of K_2_TlSbCl_6_ and K_2_TlScCl_6_ using the WIEN2k code, which is based on the FP-LAPW technique.^31^ The volume of the unit cell is centered at the Wyckoff sites: K; 8c (0.25, 0.25, 0.75), Tl: 4b (0.5, 0, 0), Sb (0, 0, 0), Sc: (0, 0, 0), and Cl: (0.23400, 0, 0). The employed FP-LAPW scheme divides the region of the unit cells into two: interstitial and muffin tin. Spherical harmonics identical to atomic orbitals are found in the muffin tin zone, while plane wave subshells expand the potential in the interstitial region.^27,28^1
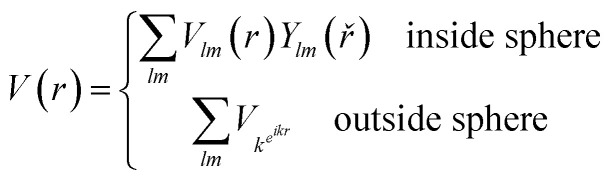


We used *R*_MT_ × *K*_max_ = 7, cut off energy = −0.6 Ry and *k*-points = 10 × 10 × 10 as input for the convergence of calculations in the first Brillouin zone. Optical characteristics were computed with a denser mesh of 2000 *K*-points. The self-consisted field were converged up to 10^−5^ Ry, and angular momentum *l*_max_ = 7 was used.^27^ The core and valence basis set were utilized as K (4s^1^), Tl (4f^14^ 5d^10^ 6s^2^ 6p^1^), Sb (4d^10^ 5s^2^ 5p^3^), Sc (4s^2^ 3d^1^), and Cl (3 s^2^ 3p^5^). Optical features were determined using the Kramers–Kronig equations, and TE features were found using the Boltz-Trap algorithm based on the semi-classical theory.^32^

## 3. Results and discussions

### 3.1 Structural properties

**Fig. 1:**
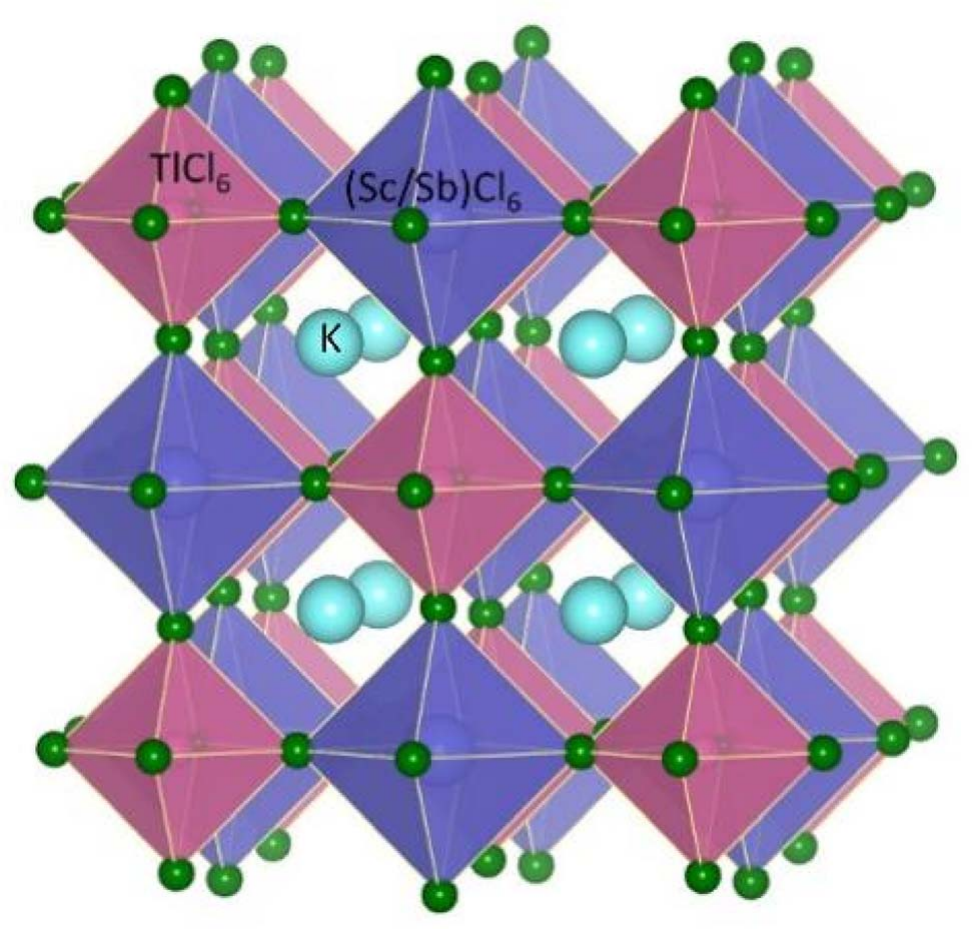
Octahedral configuration of the K_2_TlSbCl_6_ and K_2_TlScCl_6_ halide double perovskites.

**Fig. 2:**
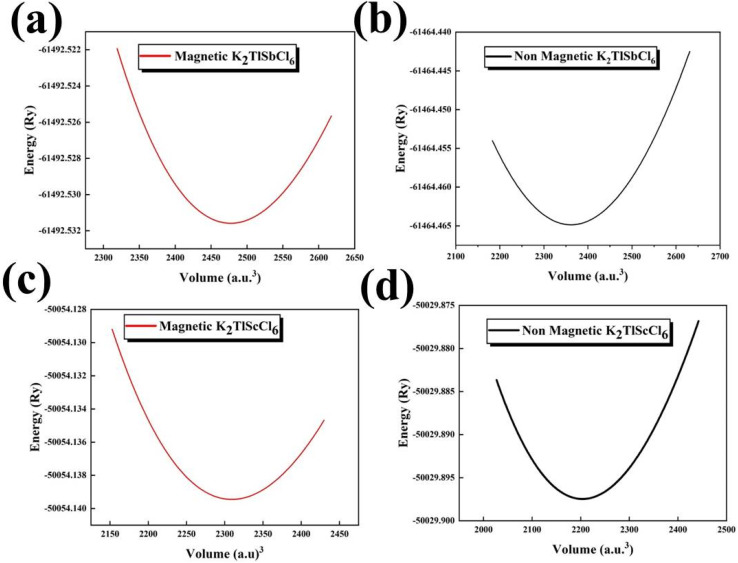
Optimized energy–volume curves of the (a and b) K_2_TlSbCl_6_ and (c and d) K_2_TlScCl_6_ halide double perovskites.

K_2_TlSbCl_6_ and K_2_TlScCl_6_ stabilize in a perovskite geometry belonging to the *Fm*3̄*m* space group (Fig. 1 and Fig. 2).^33^ The optimized crystal structures and corresponding energy–volume (*E*–*V*) plots for K_2_TlSbCl_6_ and K_2_TlScCl_6_ are presented in Fig. 2. Both nonmagnetic (NM) and ferromagnetic (FM) orderings were considered during structural optimization (Fig. 2). Δ*E* = *E*_NM_ − *E*_FM_ derived from the *E*–*V* plots confirms the thermodynamic preference for the NM phase in both compounds. Ground-state geometrical attributes were computed by fitting the total energy to the Birch–Murnaghan isothermal equation of state (EOS).^34,35^2
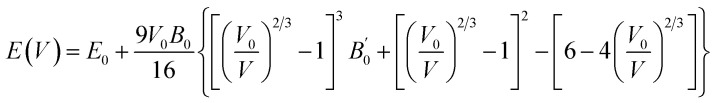


The values of the structurally optimized variables, including the lattice parameter (*a*_0_), volume (*V*_0_), bulk-moduli (*B*_0_) and its pressure derivatives 
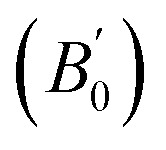
, and ground state equilibrium energy (*E*_0_), are tabulated in Table 1. To analyze the phase stability of K_2_TlSbCl_6_ and K_2_TlScCl_6_, the key structural parameters were thoroughly investigated using Goldschmidt's empirical criteria. In addition, the octahedral mismatch (Δ*µ*) and Bartel's tolerance factor (*τ*) are examined to obtain deeper insights into the lattice firmness.^36^ The Goldschmidt's tolerance factor (*t*_G_), octahedral factor (*μ̄*), and other related structural firmness parameters are computed using the following equations to ascertain the geometry of the two HDPs.3
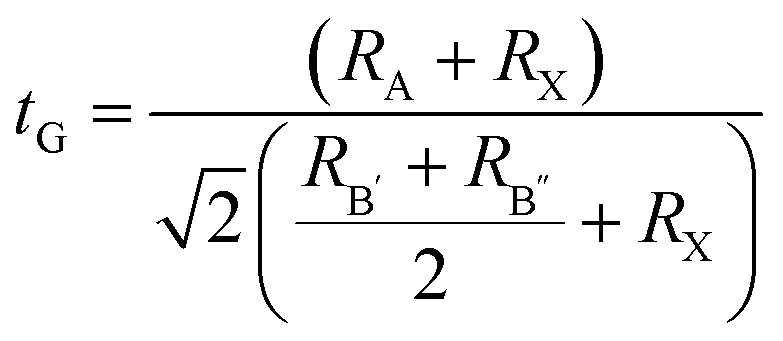
4
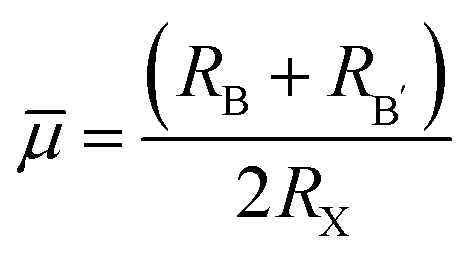
5
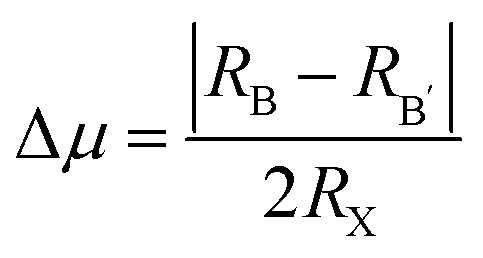
6
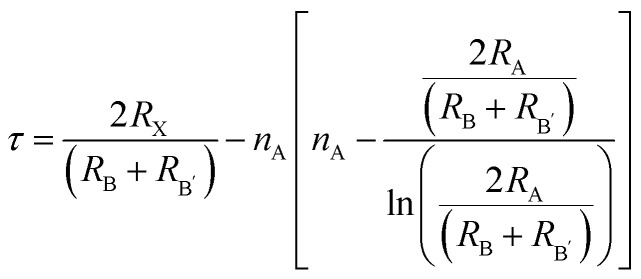
In these expressions, *R* denotes the radius of the respective ionic systems, and *n*_A_ represents the oxidation state of the A-site element. *R*_A_, *R*_X_, *R*_B′_ and *R*_B″_ are the radii of the K, Cl, Tl, and Sb/Sc ions, respectively. The *t*_G_ values for K_2_TlSbCl_6_ and K_2_TlScCl_6_ are determined to be 0.829 and 0.831, respectively, agreeing well with the cubic structure stability criteria (0.8 < *t*_G_ < 1.4).^12^ The *μ̄*, Δ*µ*, and *τ* values also fall within the cubic stability criteria. In addition to the geometrical stability analysis, thermodynamic stability analysis is presented, which can be linked with the negative value of formation energies, as computed using the equation given below.

7 Δ*H*_f_ = *E*_total_ − 2*E*_K_ + *E*_Ti_ + *E*_Sb/Sc_ + 6*E*_Cl_.

Here, *E*_total_ is the total energy of the K_2_TlXCl_6_ (X = Sb or Sc) HDPs.^24^ The computed values of Δ*H*_f_ are −2.573 and −1.929 eV for K_2_TlSbCl_6_ and K_2_TlScCl_6_, respectively (Table 1). The negative values demonstrate that the studied materials are thermodynamically stable, indicating their experimental synthesizability and potential for optoelectronic and TE applications.

**Table 1:** Calculated structural parameters, including the tolerance factor, octahedral factor, octahedral mismatch, and Bartel's tolerance factor, and thermodynamic parameter such as formation energy of the K_2_TlXCl_6_ (X = Sb or Sc) compounds

Parameter	K_2_TlSbCl_6_	K_2_TlScCl_6_
Lattice constant *a*_0_ (Å)	11.19	10.93
Bulk modulus *B*_0_ (GPa)	22.89	26.26
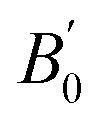	0.476	2.158
Equilibrium volume *V*_0_ (a.u.)^3^	2361.9	2203.7
Ground state energy *E*_0_ (Ryd.)	−61464.46	−50029.89
Tolerance factor (*t*_G_)	0.829	0.832
Octahedral factor (*μ̄*)	0.624	0.620
Octahedral mismatch (Δ*µ*)	0.204	0.209
Bartel's tolerance factor (*τ*)	1.193	1.206
Formation energy Δ*H*_f_ (eV)	−2.573	−1.929

### 3.2 Electronic properties

**Fig. 3:**
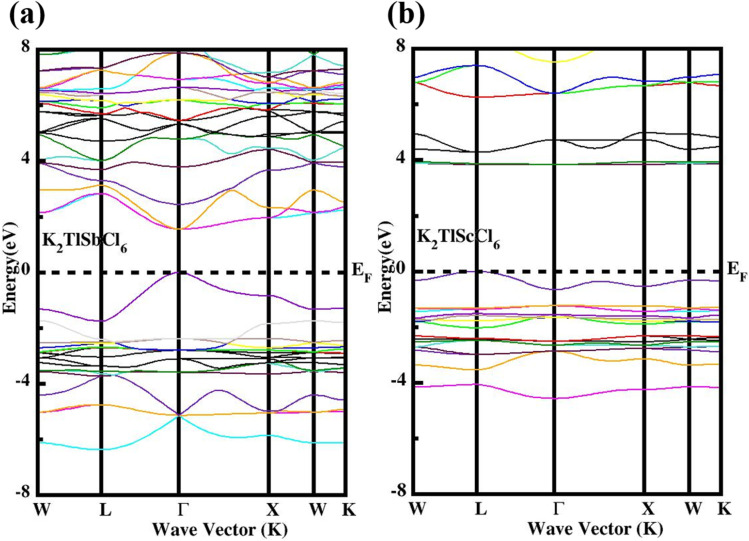
Calculated BS plots of the cubic (a) K_2_TlSbCl_6_ and (b) K_2_TlScCl_6_ halide double perovskites.

**Fig. 4:**
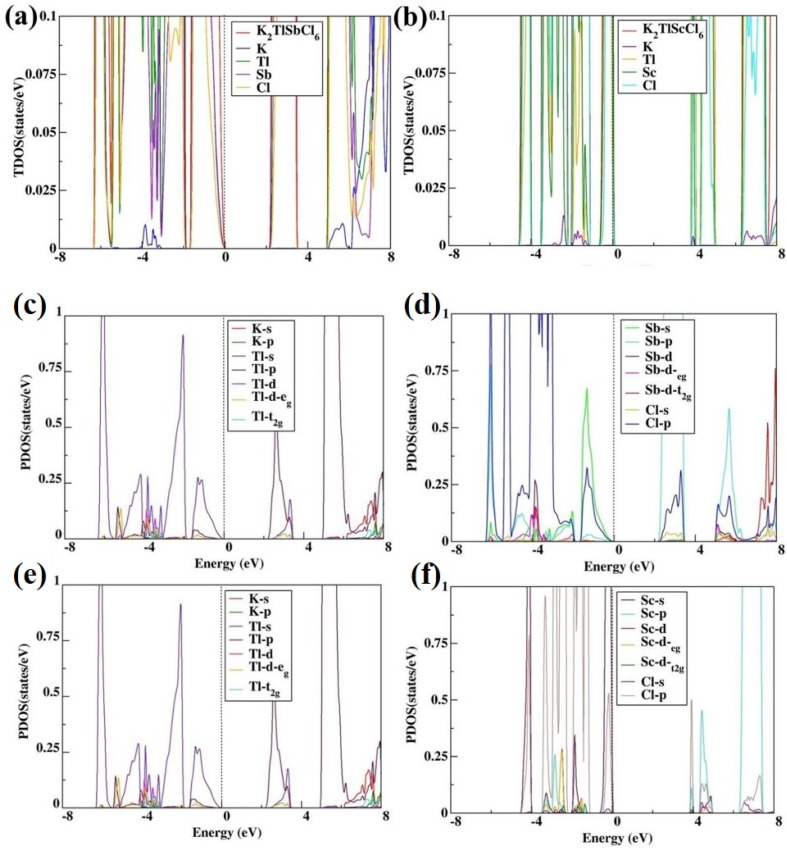
TDOS plots of (a) K_2_TlSbCl_6_ and (b) K_2_TlScCl_6_. (c and d) PDOS plots of K_2_TlSbCl_6_ and (e and f) K_2_TlScCl_6_.

The potential usage of a HDPs is strongly prompted by its electronic attributes, particularly the distribution of electrons within its band structure (BS).^37^ The generalized gradient approximation revealed a direct *E*_g_ of 3.4/1.53 eV for K_2_TlScCl_6_/K_2_TlSbCl_6_, respectively; however, these values are underestimated (SI Fig. 1S). Therefore, the mBJ potential was applied to validate the accuracy of the *E*_g_ values (Fig. 3). K_2_TlSbCl_6_ exhibits a *Γ*-centered direct *E*_g_ of 2.26 eV, revealing its semiconductive nature, while K_2_TlScCl_6_ shows an *L*-centered direct *E*_g_ of 3.83 eV. Direct *E*_g_ substances are expected to be appropriate for photon-based electronic transition devices like commercial solar energy devices.^30^ The partial (P) and total (T) density of states (DOS) were computed to comprehend the underlying electronic mechanism. The TDOS plot (see Fig. 4(a and b)) indicating peaks at energies comparable to the band dispersion plots in Fig. 3. The PDOS plots (Fig. 4c–f) show quantum level interactions between numerous sub-shells. The VB, extending from −6.1 to −2 eV, mainly consists of Cl-3p, Tl-6s, and Sc-4s subshells with a negligible influence of the Tl-5d, Tl-dt_2_g, Sc-3d, and Sb-5p orbitals. The conduction band (CB), within 2–3.94 eV, shows significant dispersions, primarily due to contributions from the Cl-3p, Tl-6s, and Sb-5p states. In the higher energy region (5 to 8 eV), the primary dispersion originates from the K-3s, K-2p, and Tl-d-t_2_g orbitals. Interestingly, K atoms play a very small role in the shallow energy levels in the VB and CB regions. Furthermore, the PDOS plots in Fig. 4(d and f) clearly show a crystal field splitting of the transition of metal d-orbitals into t_2g_ and e_g_ manifolds. This splitting results from the octahedral coordination of B-site cations (*e.g.*, Tl, Sb, or Sc) surrounded by halide anions. Specifically, the t_2g_ orbitals are oriented between the ligand axes and experience weaker electrostatic repulsion from the halide ions, resulting in a lower energy. In contrast, the e_g_ orbitals point directly toward the ligands and are destabilized to a higher energy.

### 3.3 Optical properties

**Fig. 5:**
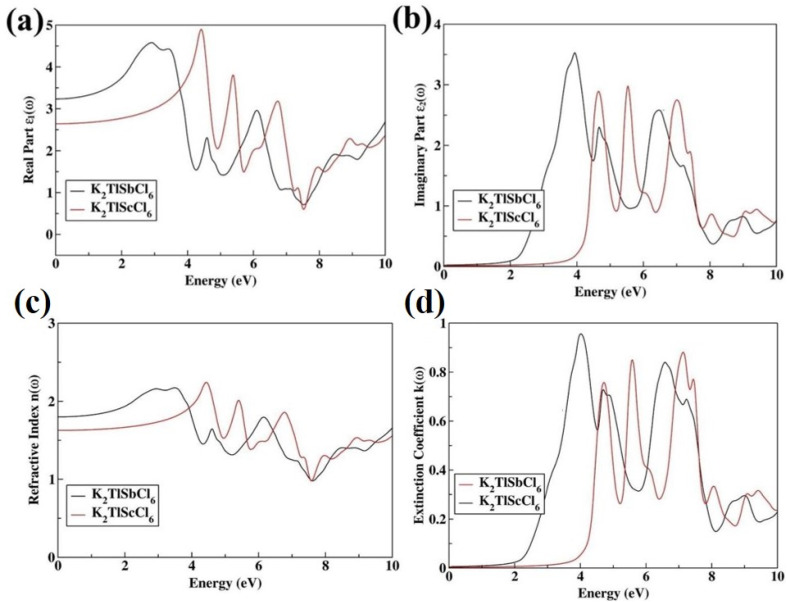
(a) *ε*_1_(*ω*), (b) *ε*_2_(*ω*), (c) *n*(*ω*) and (d) *k*(*ω*) plots of K_2_TlSbCl_6_ and K_2_TlScCl_6_.

Based on the semiconducting nature of the K_2_TlSbCl_6_ and K_2_TlScCl_6_ HDPs, we proceeded further to compute their optical response to incident light. The values of the intended dielectric tensor (real part: *ε*_1_(*ω*) and imaginary part: *ε*_2_(*ω*)) are given in Fig. 5(a and b). The concept of light polarization is explained by *ε*_1_(*ω*), while electronic transitions are linked to *ε*_2_(*ω*), which represents the material's optical absorbance.^38^ An analysis of the *ε*_1_(*ω*) spectrum shows that the *ε*_1_(0) value for K_2_TlSbCl_6_ and K_2_TlScCl_6_ are 3.25 and 2.67, respectively (Table 2). As shown by the Penn model, *ε*_1_(0) ≈ 1 + (*ħω*_p_/*E*_g_)^2^, the *ε*_1_(0) and the optical *E*_g_ are correlated.^39^

**Table 2:** Calculated static real parts of the dielectric function, reflectivity, and refractive index of the K_2_TlXCl_6_ (X = Sb or Sc) compounds

Optical parameter	K_2_TlSbCl_6_		K_2_TlScCl_6_
	Current	Previous	
*ε*_1_ (0)	3.25	3.59,^27^ 3.18 (ref. 30) and 2.24 (ref. 31)	2.67
*n* (0)	1.82	1.89 (ref. 27) and 1.49 (ref. 31)	1.64
*R* (0)	0.08	0.09 (ref. 27) and 0.03 (ref. 31)	0.05

The higher peaks of both the K_2_TlSbCl_6_ and K_2_TlScCl_6_ compounds were found to be at 2.87 and 4.40 eV, while lower peaks occurred at 2.87 and 4.40 eV, respectively. *ε*_1_(0) can be computed through the Kramers–Kronig relations.^6,11^8
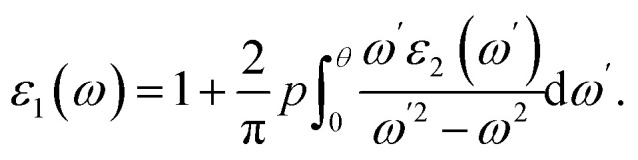
In the *ε*_2_(*ω*) plot, the transitions of electrons from the VB to CB are illustrated, providing a measure of the materials' optical absorption, and it is entirely associated with the BS,^40^ (see Fig. 5(b)). The threshold energy values for investigated materials are revealed by the *ε*_2_(*ω*) spectrum, which corresponds to the band dispersion plots. The lower peaks of both the K_2_TlSbCl_6_ and K_2_TlScCl_6_ compounds were found at 8.10 and 8.70 eV, while the higher peaks occurred at 3.94 and 5.52 eV, respectively. The *ε*_2_(*ω*) part is computed by the following equation:^31^9
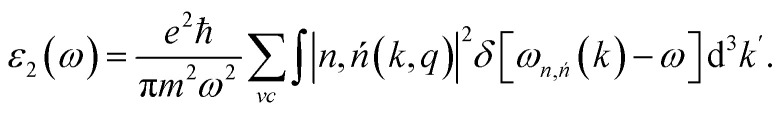


An essential physical parameter in optics is the refractive index *n*(*ω*), which can be computed using the following expression:^41^10
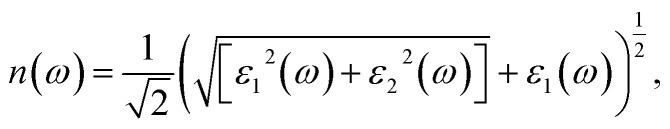
while the static *n*(0) can be computed as^42^11
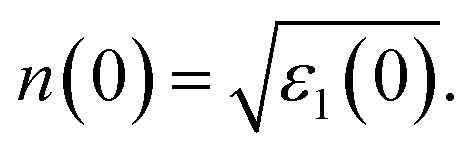
*n*(*ω*) characterizes the ability of a substance to bend light, making it a critical factor for determining the desirable compounds for solar cells, optoelectronic detectors and waveguides.^43^ The values of the static refractive indices *n*(0) of both the K_2_TlSbCl_6_ and K_2_TlScCl_6_ compounds were 1.82 and 1.64, respectively (Table 2). A minimum of *n*(*ω*) was found at 7.68 for K_2_TlSbCl_6_ and at 7.54 eV for K_2_ScTlCl_6_ while the maxima appeared at 3.5 and 4.43 eV for K_2_TlSbCl_6_ and K_2_ScTlCl_6_, respectively, as illustrated in Fig. 5c. The *k*(*ω*) is the complex part of the index that tells us the feasibility of the electromagnetic waves to pass through any material. *ε*_2_(*ω*) is linked with *k*(*ω*), which regulates the transmission of electromagnetic waves through a substance. It can be obtained with the following equation:^44^12
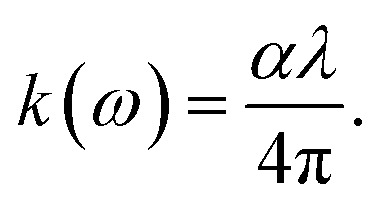


The *k*(*ω*) plot shows the minima of the compounds at 8.15 and 8.72 eV, while the maxima of the two compounds occur at 4.02 and 7.12 eV for K_2_TlSbCl_6_ and K_2_TlScCl_6_, respectively (Fig. 5d). For solar cells and related technologies, reflectivity is an essential optical property. Both the examined HDP materials exhibit low *R*(*ω*) in the visible-UV region. Reflectivity *R*(*ω*) can be computed as follows:^45^13
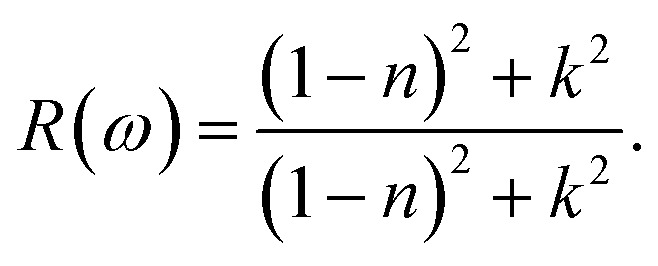


**Fig. 6:**
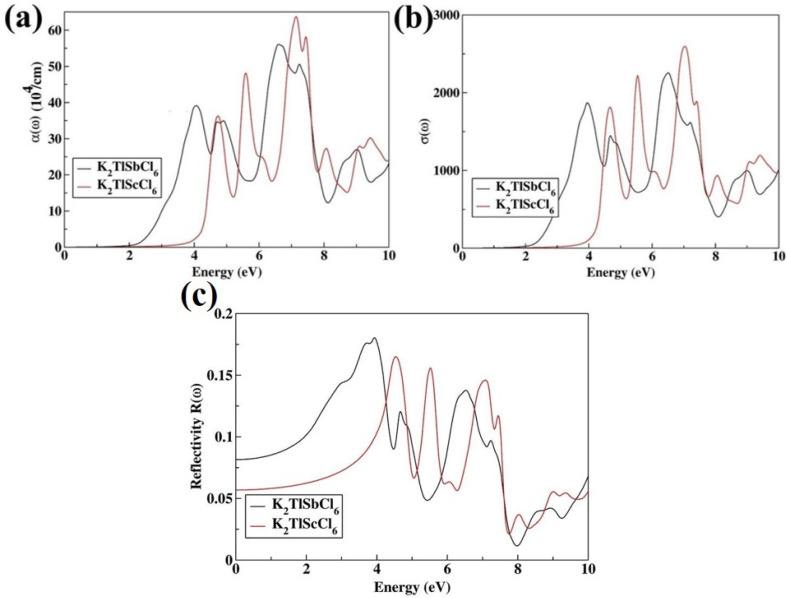
(a) *α*(*ω*), (b) *σ*(*ω*) and (c) *R*(*ω*) plots of K_2_TlSbCl_6_ and K_2_TlScCl_6_.

The static reflectivity of K_2_TlSbCl_6_ and K_2_TlScCl_6_ were 0.08 and 0.05 (Table 2), respectively. The minima of *R*(*ω*) was found at 8.00 for K_2_TlSbCl_6_ and 7.74 eV for K_2_TlScCl_6_, while the maxima appeared at 3.94 and 4.54 eV for K_2_TlSbCl_6_ and K_2_TlScCl_6_, respectively (Fig. 6c). Another two important optical parameters are the coefficient of optical absorption *α*(*ω*) and optical conduction *σ*(*ω*). *α*(*ω*) is the measure of light absorbance per unit length deep inside the material that is directly linked with *ε*_2_(*ω*) and can be represented as follows:^46^14
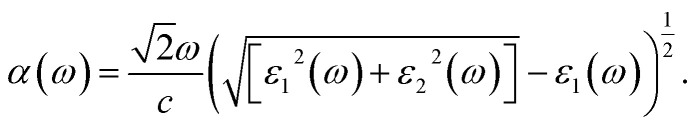


Calculated absorption coefficients demonstrate minimal peaks at 8.13/5.19 eV, while maximal absorption peaks are observed at 6.59/7.13 eV for K_2_TlSbCl_6_ and K_2_TlScCl_6_ (Fig. 6a), respectively. Optically active free electrons are produced in a material when it absorbs incident light. These electrons enhance the conduction of a given substance, and this phenomenon is called optical conductivity *σ*(*ω*), which can be obtained using the following relation:^47^15
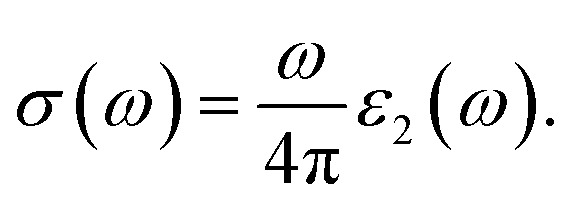


The minima of *σ*(*ω*) were found at 7.95 for K_2_TlSbCl_6_ and 5.16 eV for K_2_TlScCl_6_ while the maxima appeared at 6.50 and 7.02 eV for K_2_TlSbCl_6_ and K_2_TlScCl_6_, respectively (Fig. 6b). The maximum absorption coefficient and *σ*(*ω*) of both the compounds and the lower reflectivity confirm the use of the titled materials in UV optoelectronic devices.

### 3.4 Elastic properties

Elastic properties describe the mechanical behavior of a material, including its stiffness, ductility, strength, flexibility and resistance to deformation. These properties determine the material's mechanical response to applied strain and stress. In cubic crystal systems, mechanical stability is determined by three elastic constants: *C*_11_, *C*_12_, and *C*_44_.^48^*C*_11_ reflects the resistance of a material to longitudinal strain, *C*_12_ represents the elastic coupling between stresses along different axes, and *C*_44_ measures the resistance against shape distortion. The stability of K_2_TlSbCl_6_ and K_2_TlScCl_6_ is assessed using Born and Huang stability criteria: *C*_44_ > 0, *C*_11_ − *C*_12_ > 0, *C*_11_ + 2*C*_12_ > 0, *C*_11_ > 0, and *C*_12_ < *B* < *C*_11_.^49^

The computed elastic constants enable the determination of several mechanical properties, including the Pugh's ratio (*B*/*G*), Cauchy pressure (*C*_P_), bulk modulus (*B*), shear modulus (*G*), anisotropic factor (*A*), Poisson's ratio (*ν*) and Young's modulus (*E*). These parameters are evaluated through the Voigt–Reuss–Hill (VRH) scheme.^26,50^16
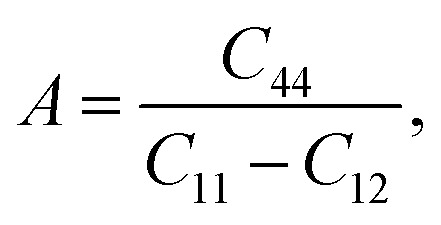
17
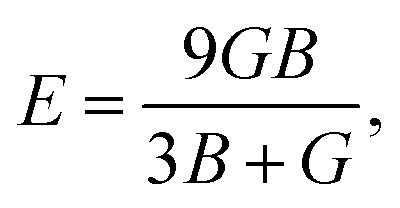
18
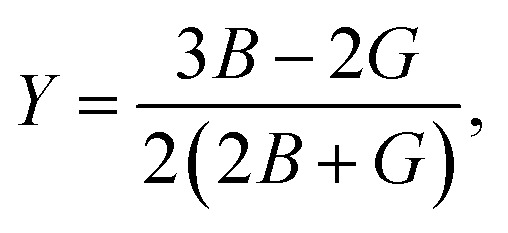
19
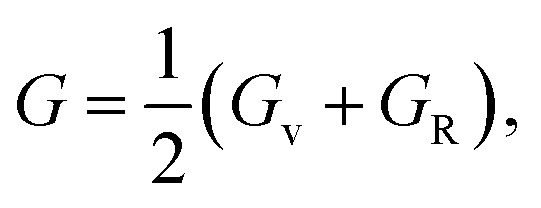
20
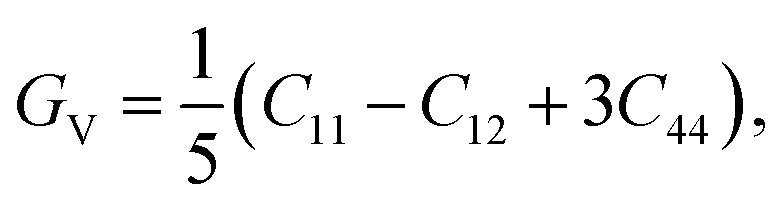
21
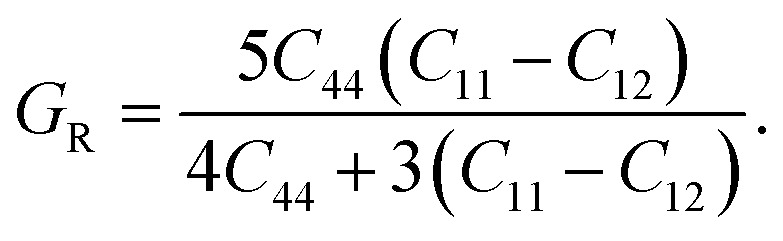


**Fig. 7:**
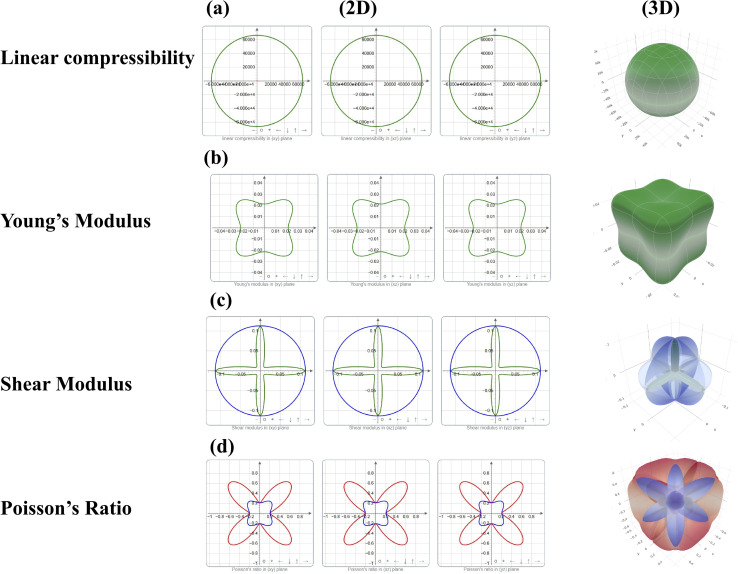
2D and 3D representations of the elastic moduli of K_2_TlSbCl_6_.

**Fig. 8:**
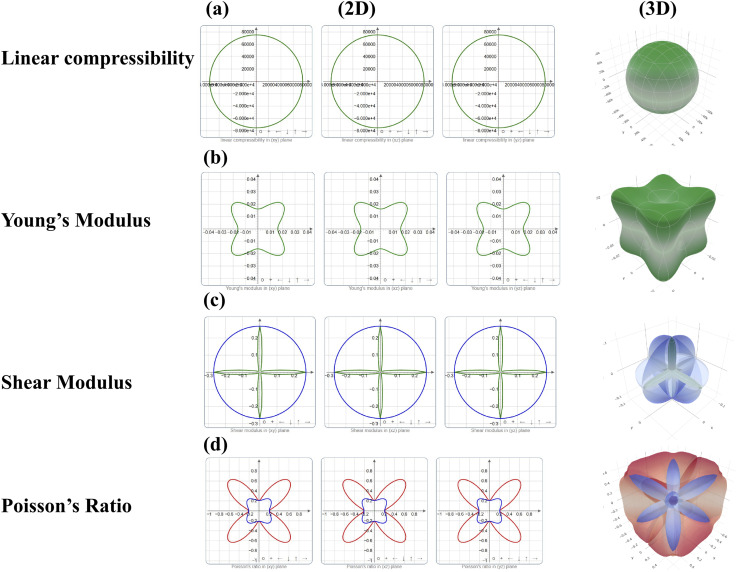
2D and 3D representations of the elastic moduli of K_2_TlScCl_6_.

*B* reflects the ability of a material to resist uniform compression. The calculated results suggest that the compounds can tolerate both volume and shape distortions while maintaining compressibility. The *B* of K_2_TlScCl_6_ (25.07) exceeds that of K_2_TlSbCl_6_ (22.12), indicating that K_2_TlScCl_6_ is more resistant to volume distortion. *E* describes the stiffness of a material under applied stress. The computed values of *E* are 30.43 for K_2_TlSbCl_6_ and 25.34 for K_2_TlScCl_6_, depicting that K_2_TlSbCl_6_ has greater stiffness than K_2_TlScCl_6_. *G* illustrates a material's response to shape distortion; the *B*/*G* ratio provides the evidence for whether a material is brittle/ductile. The critical value of the *B*/*G* ratio is 1.75; values lower than this threshold indicate brittle behavior while higher values suggest ductility.^51^ Both compounds exhibit ductile behavior (see Table 3). Furthermore, Cauchy pressure (*C*_P_ = *C*_12_ − *C*_44_) and Poisson's ratio (*ν*) provide insights into the ductile or brittle nature of materials. A positive *C*_P_ value suggests ductility, while a negative *C*_P_ value suggests brittleness. The positive *C*_P_ values of both materials further support their ductility. According to the criterion proposed by I. N. Frantsevich, materials with *ν* > 0.26 exhibit a ductile behavior while those with *ν* < 0.26 reveal ductile feature.^52^ The calculated *ν* ratios reported in Table 3 further indicate the ductile character of the investigated materials. The anisotropy factor describes the directional variation in the elastic properties of a material. The value of *A* = 1 reveals the isotropic behavior and any deviation indicates anisotropy.^53^ The computed values are 0.27 for K_2_TlSbCl_6_ and 0.33 for K_2_TlScCl_6_, signifying elastic anisotropy. The anisotropic behavior of *G*, linear compressibility, *ν* and *E* are illustrated in Fig. 7(a–d) and 8(a–d) through both 2D and 3D representations. Linear compressibility (*β*) describes the directional strain response of a material under uniaxial stress, indicating expansion or contraction along specific crystallographic directions. Such directional mechanical characteristics are important for assessing the material's firmness and performance of solar cell materials.^54^ In an ideal isotropic system, elastic properties exhibit spherical symmetry, reflecting invariance with respect to direction. Deviations from perfect symmetry are indicative of elastic anisotropy.^55^

**Table 3:** Calculated elastic parameters of the K_2_TlXCl_6_ (X = Sb or Sc) compounds

Parameter	K_2_TlSbCl_6_	K_2_TlScCl_6_
*G* (GPa)	11.98	9.51
*B* (GPa)	22.13	25.07
*E* (GPa)	30.43	25.34
*B*/*G*	1.847	2.634
*N*	0.272	0.33
*A*	0.473	0.134
*C*_11_ (GPa)	46.94	62.11
*C*_12_ (GPa)	9.72	6.55
*C*_44_ (GPa)	8.84	3.72

As observed in Fig. 7 and 8, both K_2_TlScCl_6_ and K_2_TlSbCl_6_ display noticeable deviations from spherical symmetry, confirming their anisotropic nature. In contrast, linear compressibility retains a circular distribution, indicating isotropic behavior. This suggests that despite significant directional dependence in most elastic properties, linear compressibility remains isotropic in these cubic systems. The degree of anisotropy is further confirmed by the extreme values of *ν* and *G*, as summarized in Table 4.

**Table 4:** Minimum and maximum values of the elastic moduli, along with their corresponding anisotropy ratios, for the K_2_TlXCl_6_ (X = Sb or Sc) compounds

Compound	Linear compressibility (GPa^−1^)			Young's modulus (GPa)			Shear modulus (GPa)			Poisson's ratio		
	*β*_min_	*β*_max_	*A*	*E*_min_	*E*_max_	*A*	*G*_min_	*G*_max_	*A*	*ν*_min_	*ν*_max_	*A*
K_2_TlSbCl_6_	0.01505	0.0150	1	28.5	43.6	1.53	8.85	17.8	2.01	0.17	0.33	1.94
K_2_TlScCl_6_	0.01329	0.0132	1	21.7	60.8	2.80	3.72	7.41	1.99	0.096	0.42	4.37

Quantitative analysis of the elastic parameters reveals distinct differences between the two compounds. K_2_TlSbCl_6_ exhibits moderate anisotropy, as evidenced by the Young's modulus ranging from 28.5 to 43.6 GPa (*A* ≈ 1.53) and shear modulus from 8.85 to 17.8 GPa (≈2.01). In comparison, K_2_TlScCl_6_ shows a significantly broader variation in its Young's modulus (21.7–60.8 GPa), corresponding to a higher anisotropy ratio (≈2.80), indicative of enhanced stiffness anisotropy. However, its shear modulus variation (3.72–7.41 GPa; ≈1.99) remains comparable to that of K_2_TlSbCl_6_. Poisson's ratio exhibits substantial directional variation, ranging from 0.17 to 0.33 (≈1.94) for K_2_TlSbCl_6_ and from 0.096 to 0.42 (≈4.37) for K_2_TlScCl_6_, reflecting stronger anisotropic lateral deformation in the latter. Meanwhile, linear compressibility remains isotropic (*A* = 1) in both compounds, consistent with their cubic symmetry (Table 5).

**Table 5:** Calculated thermoelectric parameters: electrical conductivity, thermal conductivity, Seebeck coefficient, power factor, and figure of merit of the K_2_TlXCl_6_ (X = Sb or Sc) compounds

Thermoelectric parameter	K_2_TlSbCl_6_ (K)		K_2_TlScCl_6_ (K)	
	300	800	300	800
Electrical conductivity (*σ*/*τ*) × 10^19^ (Ω^−1^ m^−1^ s^−1^)	0.023	0.09	0.58	1.02
Thermal conductivity (*k*_e_/*τ*) × 10^14^ (W m^−1^ K^−1^ s^−1^)	0.049	0.570	0.739	2.885
Seebeck coefficient (*S*) × 10^−5^ (µV K^−1^)	236.90	242.96	150.76	159.58
Power factor (*S*^2^*σ*/*τ*) × 10^11^ (µW K^−2^ cm^−1^)	0.128	0.534	1.329	2.616
*ZT* = ((*S*^2^ × *σ*/*κ*) × *T*)	0.78	0.75	0.54	0.73

### 3.5 Thermoelectric (TE) properties

As elaborated in the introduction section, fossil fuel consumption has increased dramatically in last few decades, mainly due to the outstanding growth of the global economy, resulting in significantly severe environmental damage and energy shortages.^56–58^ Since TE materials have the ability to directly convert heat into electrical power, they have attracted significant research interest.^59^ We computed the TE parameters between 300 and 800 K using the BoltzTraP algorithm.^60^ The BS performs a key role in determining the TE parameters as these are primarily influenced by the effective mass, concentration, and type of charge carriers. Moreover, the BS around the *E*_f_ are closely associated with the material's transport behavior.

**Fig. 9:**
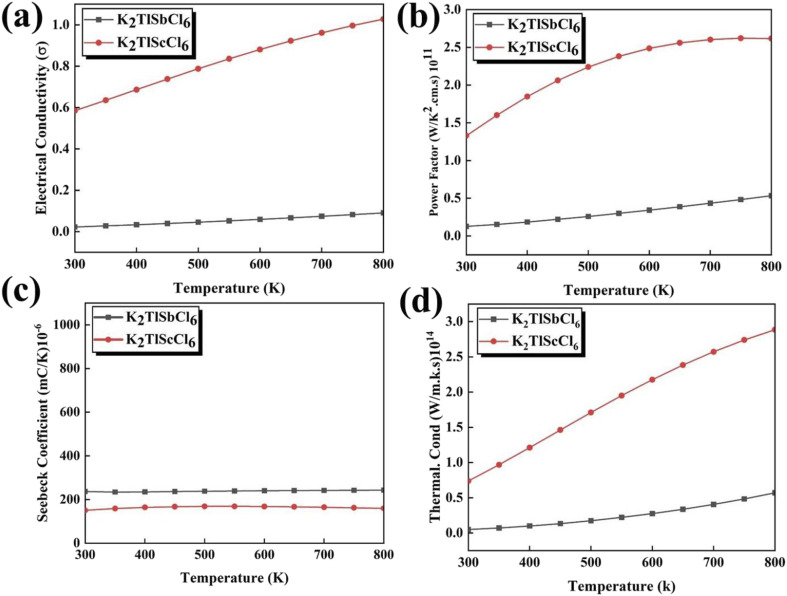
*σ*/*τ* (a), PF (b), *S* (c) and *k*/*τ* (d) plots of K_2_TlSbCl_6_ and K_2_TlScCl_6_.

The number of accessible electrons *via* conduction is calculated by the ratio *σ*/*τ*. It is found that *σ*/*τ* rises with temperature. Its value at 300 K is 0.023 × 10^−19^ for K_2_TlSbCl_6_ and 0.584 × 10^−19^ (Ω^−1^ m^−1^ s^−1^) for K_2_TlScCl_6_. It is observed that when Sc is used in place of Sb, *σ*/*τ* increases. At 800 K, it is noted that K_2_TlSbCl_6_ has a peak value of 0.09 × 10^−19^, while K_2_TlScCl_6_ has the highest value of 1.03 × 10^−19^ (Ω^−1^ m^−1^ s^−1^) (Fig. 9a). The investigated materials are perfect for TE appliances since both offer the high electrical conductivities. The ratio *k*/*τ* is the amount of heat transference from one point to another in a material. It has two parts: (i) electronic conductivity (*K*_e_) and lattice conductivity (*K*_l_). Both electrical and photonic components are involved in the thermal conductivity (*k*_e_/*τ*) in semiconductor substances. However, at temperatures greater than 300 K, *K*_l_ values diminish nearly to zero. The predicted *k*_e_/*τ* values for K_2_TlSbCl_6_ and K_2_TlScCl_6_ at 300 K are 0.049 and 0.74, while the values at 800 K are 0.57 and 2.88 (Wm^−1^ K^−1^ s^−1^), respectively (Fig. 9d), which represents an increasing trend with temperature. This shows good correspondence with the Wiedemann–Franz law, which correlates electrical and thermal conductivities and is expressed as *κ* = *σLT*.^61^

A temperature difference generates thermal electromotive forces that produce a potential difference of several microvolts, known as the Seebeck effect. The formula *S* = Δ*V*/Δ*T* is used to assess the Seebeck coefficient.^62^ Band dispersions, particularly near the *E*_f_, are strongly linked to the magnitude of *S*, whose dependence on effective mass and carrier concentration is given by 
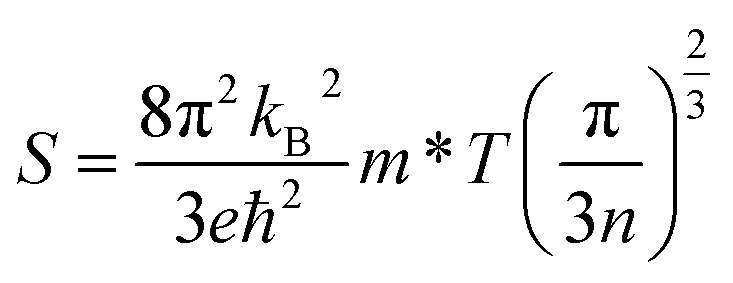
. Here, m* denotes the effective mass and *n* denotes the carrier concentration.^63^ The *S* values for K_2_TlSbCl_6_ and K_2_TlScCl_6_ are 236.9 and 150.7 µV K^−1^ at 300 K and 242.9 and 159.6 µV K^−1^ at 800 K (Fig. 9c). The value of *S* decreases with an increase in temperature and is proportional to *σ*/*τ* for both materials. Such large *S* values are achievable only when the material possesses a relatively high carrier concentration. Moreover, the consistently positive *S* values indicate that the studied materials retain the p-type semiconductor behavior across the entire temperature range.

PF serves as a key indicator of efficiency as it essentially predicts the power output of TE devices, and it can be computed as PF = *σS*^2^. The maximum values of PF for K_2_TlSbCl_6_ and K_2_TlScCl_6_ at 800 K are 0.53 × 10^11^ and 2.62 × 10^11^ W K^−2^ s^−1^, respectively (Fig. 9b). Owing to their high *S* values, the investigated materials exhibit a significantly enhanced PF.

The *ZT* parameter is one of the most crucial factors in forecasting the operation of TE devices and can be computed as follows:^64,65^22
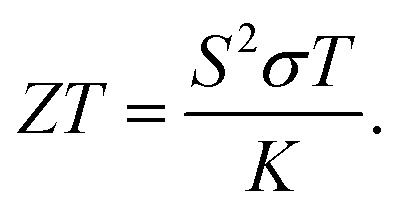


**Fig. 10:**
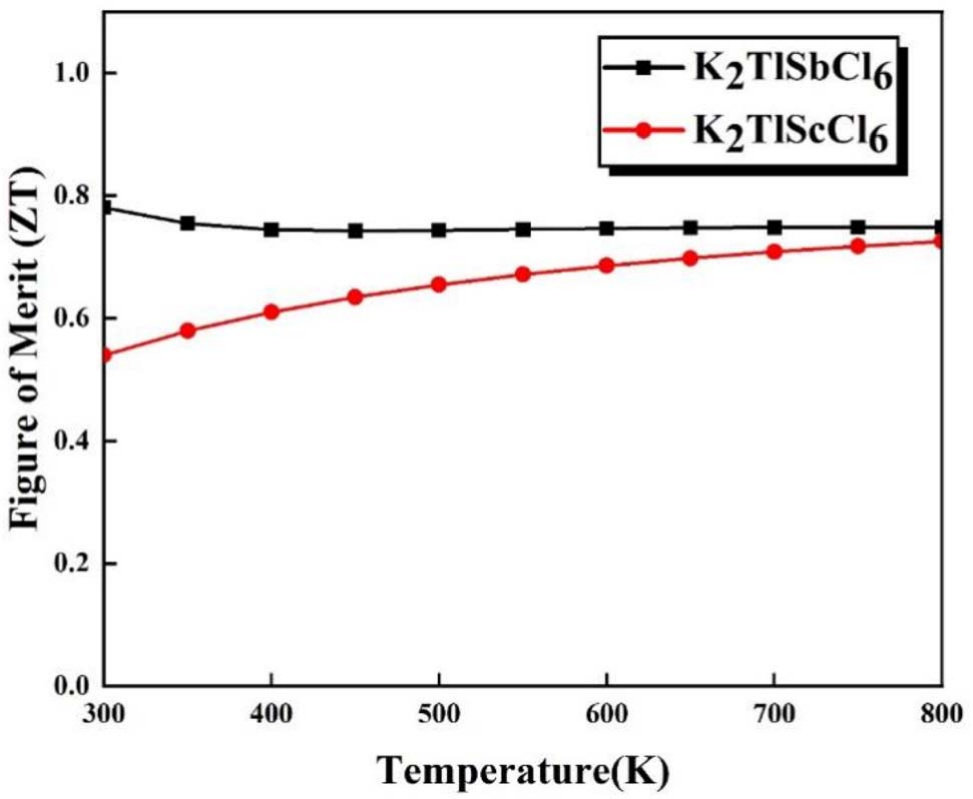
*ZT* plots of K_2_TlSbCl_6_ and K_2_TlScCl_6_.

The lower *σ*/*τ* of the Sb-based HDP and the higher *σ*/*τ* of the Sc-based HDP influence the efficiency of TE devices. The *ZT* values for K_2_TlSbCl_6_ and K_2_TlScCl_6_ at 800 K are 0.75 and 0.73, respectively (Fig. 10). Overall, K_2_TlSbCl_6_ and K_2_TlScCl_6_ exhibit remarkable TE properties, indicating their potential as promising materials for TE coolers and generators.

### 3.6 Photocatalytic properties

The global energy crisis and increasing environmental concerns have accelerated the research on efficient photocatalytic materials for sustainable energy applications. Photocatalysis has emerged as an effective pathway for renewable energy production by converting sunlight directly into hydrogen. In this regard, perovskite-based materials have gained substantial interest because of their tunable lattice, adjustable band gaps, variable oxidation and valence states, and versatile compositions.^66^

The *E*_g_ of a semiconducting material plays a crucial part in finding its photocatalytic activity. An optimal photocatalyst possesses a *E*_g_ greater than 1.23 eV to facilitate both the oxygen and hydrogen evolution reactions but smaller than 3.0 eV to allow efficient solar light absorption. Furthermore, the band edges must align with the redox potentials of water. On the vacuum energy scale, the conventional water oxidation and reduction potentials, corresponding to O_2_/H_2_O and H^+^/H_2_, are −5.64 eV and −4.44 eV, respectively. The CB edge (*E*_CBM_) must exceed the H^+^/H_2_ reduction potential (−4.44 eV) to derive photo-excited electrons from VB to produce H_2_. In contrast, the VB edge (*E*_VBM_) should remain below the O_2_/H_2_O (−5.64 eV) oxidation potential to ensure smooth hole transfer for oxygen evolution. For the studied materials, the CB condition is satisfied, while the VB requirement is assessed *via* Mulliken's electronegativity.^67^23
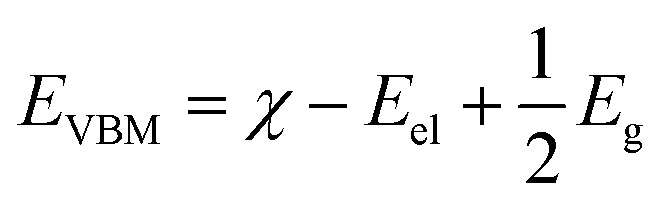

24 *E*_CBM_ = *E*_VBM_ − *E*_g_

**Fig. 11:**
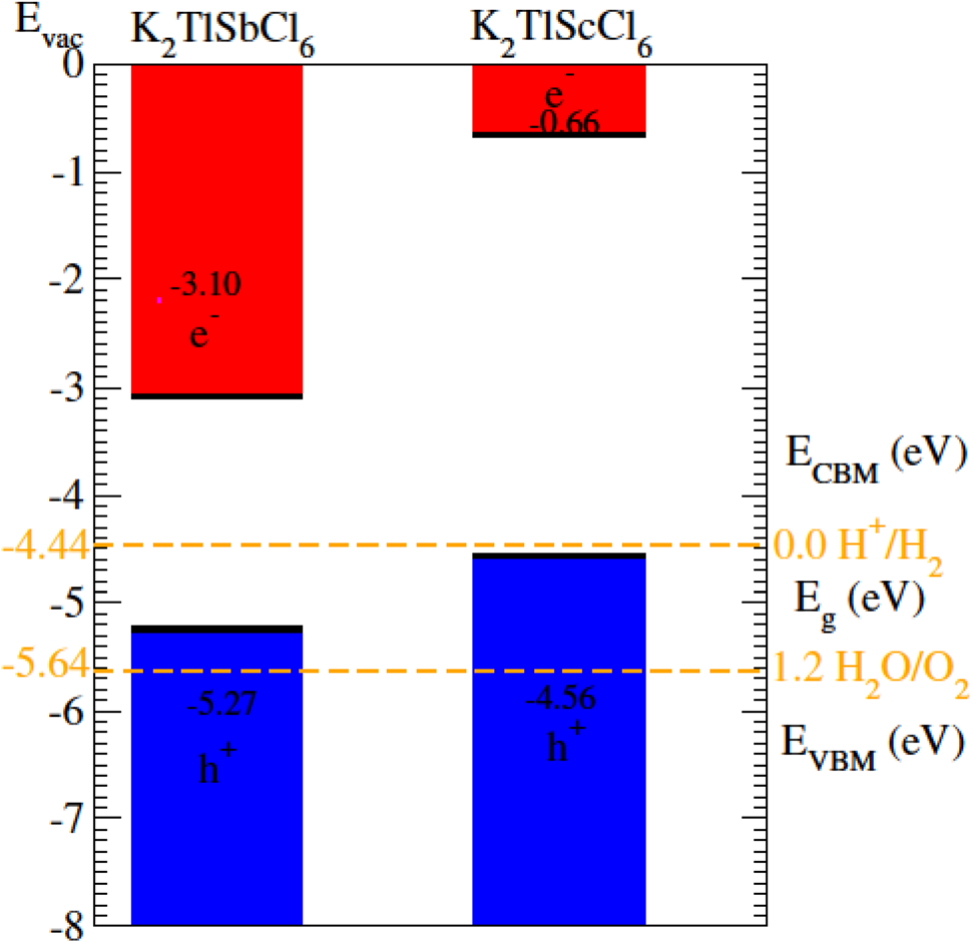
Computed band-edge alignment of the K_2_TlSbCl_6_ and K_2_TlScCl_6_ halide double perovskites.

The standard electrode potential on the hydrogen scale is taken as *E*_el_ = 4.5 eV. Here, *χ* denotes Mulliken's electronegativity, calculated as 
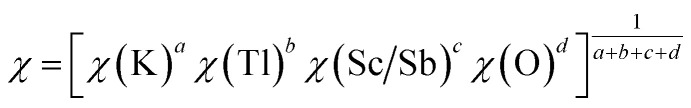
 and *a*, *b*, *c*, and *d* are the number of individual atoms present in the compound. *χ*(*x*) is computed using the electron affinity (*E*_EA_) and first ionization potential (*E*_IE_) of the corresponding atom 
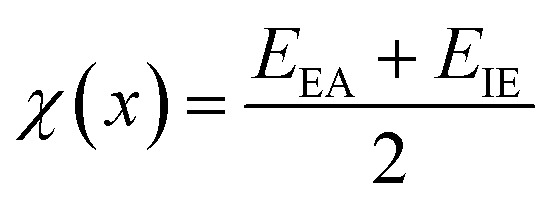
. Fig. 11 illustrates the band-edge alignment of the investigated K_2_TlXCl_6_ (X = Sc and Sb) compounds. For K_2_TlSbCl_6_, the CBM is positioned at −3.10 eV, which is slightly more negative than the H^+^/H_2_ reduction potential on the vacuum scale. Consequently, the photogenerated electrons possess insufficient reducing power to drive hydrogen evolution. Similarly, its VBM lies at −5.27 eV, which is less negative than the H_2_O/O_2_ oxidation potential (−5.64 eV), indicating its insufficient oxidizing ability for oxygen evolution. Therefore, K_2_TlSbCl_6_ is not thermodynamically promising for overall water splitting.

In contrast, K_2_TlScCl_6_ indicates the CBM at −0.66 eV, which provides a substantial thermodynamic driving force for proton reduction and hydrogen evolution. However, its VBM is positioned at −4.56 eV, which is significantly less negative than the H_2_O/O_2_ oxidation potential, indicating its inadequate oxidizing power for water oxidation. The analysis of band-edge alignment indicates that K_2_TlScCl_6_ and K_2_TlSbCl_6_ are not potentially suitable for overall water splitting as they both fail to meet the valence-band requirement for the oxygen evolution reaction. However, K_2_TlScCl_6_ fulfills the conduction-band criterion and hence is suitable for hydrogen generation.

## 4. Conclusion

Based on the first-principles calculations, K_2_TlXCl_6_ (X = Sb or Sc) are predicted to be wide-*E*_g_ nonmagnetic semiconductors. The calculated *t*_G_ and formation enthalpy values verify the lattice and thermodynamic firmness of both HDPs in cubic phases. Both HDPs fulfill the Born benchmark. Notably, K_2_TlScCl_6_ exhibits a greater stiffness with *C*_11_ = 62.11 GPa as compared with K_2_TlSbCl_6_, which has *C*_11_ = 46.94 GPa. Electronic properties revealed that K_2_TlScCl_6_ and K_2_TlSbCl_6_ exhibit a direct *E*_g_ of 3.83 and 2.26 eV, respectively, which highlight the significance of these HDPs for a range of high-frequency photonic applications. The optical features show a high *α*(*ω*) of ≈6.59 × 10^5^ cm^−1^ for K_2_TlSbCl_6_ and 7.13 × 10^5^ cm^−1^ for K_2_TlScCl_6_, paired with low reflectivity. Moreover, temperature-dependent TE characteristics reveal a higher TE efficiency with *ZT* values higher than 0.7. The present first-principles characterization identifies K_2_TlXCl_6_ (X = Sb or Sc) as highly stable double perovskites with strong potential for further experimental investigations, which may ultimately enable advanced technological applications.

## Conflicts of interest

There are no conflicts to declare.

## Supplementary Material

RA-OLF-D6RA03926J-s001

## Data Availability

Data will be available from the authors upon reasonable request. Supplementary information (SI): calculated BS for cubic (a) K_2_TlSbCl_6_ and (b) K_2_TlScCl_6_ compounds with GGA method. See DOI: https://doi.org/10.1039/d6ra03926j.
