# Supplementary material for: First-principles investigation of the mechanically and thermodynamically stable K2TlXCl6 (X = Sb or Sc) compounds for energy harvesting and photocatalytic applications
Source: RSC Adv. 2026 Jul 8. Online ahead of print. doi: 10.1039/d6ra03926j (PMC13344879; doi:10.1039/d6ra03926j)
Supplement: RA-OLF-D6RA03926J-s001 [file RA-OLF-D6RA03926J-s001.pdf]

Supplementary Information (SI) for RSC Advances.
This journal is © The Royal Society of Chemistry 2026

Supplementary data

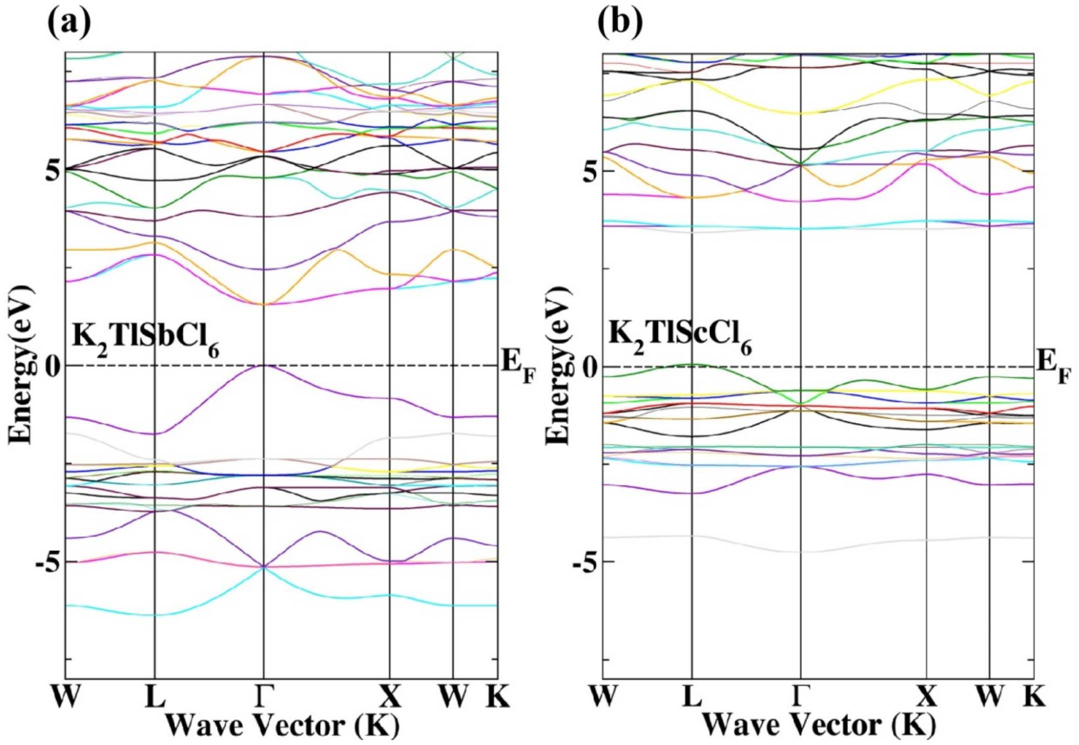

Fig. 1S Calculated BS for cubic (a) $K_2TlSbCl_6$ and (b) $K_2TlScCl_6$ compounds
